# Harmonic imaging for nonlinear detection of acoustic biomolecules

**DOI:** 10.1063/5.0214306

**Published:** 2024-11-12

**Authors:** Rohit Nayak, Mengtong Duan, Bill Ling, Zhiyang Jin, Dina Malounda, Mikhail G. Shapiro

**Affiliations:** 1 Division of Chemistry and Chemical Engineering, California Institute of Technology, Pasadena, California 91125, USA; 2 Division of Biology and Biological Engineering, California Institute of Technology, Pasadena, California 91125, USA; 3 Andrew and Peggy Cherng Department of Medical Engineering, California Institute of Technology, Pasadena, California 91125, USA; 4 Howard Hughes Medical Institute, California Institute of Technology, Pasadena, California 91125, USA

## Abstract

Gas vesicles (GVs) based on acoustic reporter genes have emerged as potent contrast agents for cellular and molecular ultrasound imaging. These air-filled, genetically encoded protein nanostructures can be expressed in a variety of cell types *in vivo* to visualize cell location and activity or injected systemically to label and monitor tissue function. Distinguishing GV signal from tissue deep inside intact organisms requires imaging approaches such as amplitude modulation (AM) or collapse-based pulse sequences. However, these approaches have limitations either in sensitivity or require the destruction of GVs, restricting the imaging of dynamic cellular processes. To address these limitations, we developed harmonic imaging to enhance the sensitivity of nondestructive GV imaging. We hypothesized that harmonic imaging, integrated with AM, could significantly elevate GV detection sensitivity by leveraging the nonlinear acoustic response of GVs. We tested this hypothesis by imaging tissue-mimicking phantoms embedded with purified GVs, mammalian cells genetically modified to express GVs, and mice liver *in vivo* post-systemic infusion of GVs. Our findings reveal that harmonic cross-propagating wave AM (HxAM) imaging markedly surpasses traditional xAM in isolating GVs' nonlinear acoustic signature, demonstrating significant (p < 0.05) enhancements in imaging performance. HxAM imaging improves detection of GV producing cells up to three folds *in vitro*, enhances *in vivo* imaging performance by over 10 dB, while extending imaging depth by up to 20%. Investigation into the backscattered spectra further elucidates the advantages of harmonic imaging. These advancements bolster ultrasound's capability in molecular and cellular imaging, underscoring the potential of harmonic signals to improve GV detection.

Ultrasound imaging plays a pivotal role in medical diagnostics, offering high spatial and temporal resolution for examining organ anatomy and function. The development of micro- and nanoscale contrast agents has extended ultrasound's reach considerably,^1^ while the introduction of acoustic reporter genes (ARGs) encoding gas vesicle (GV) proteins has broadened the capabilities of ultrasound imaging at the molecular and cellular level.^2^ Particularly, GVs are air-filled protein nanostructures, and are the first genetically encodable ultrasound reporters^3–5^—encoded by gene clusters of eight or more genes, originally evolved in various aquatic bacteria and archaea to regulate buoyancy for optimal sunlight exposure and nutrient uptake.^6,7^

GVs are distinctive in their structure: typically ∼85 nm in diameter and ∼500 nm in length, they are encapsulated by a protein shell about 3 nm thick that can endure large pressures up to hundreds of kilopascals without collapsing.^8,9^ The shell's interior is markedly hydrophobic, preventing water ingress while permitting gas molecules to freely diffuse in and out.^6,7^ The GV shell comprises a primary structural protein, GvpA—a small (7-kDa) and amphiphilic molecule that polymerizes to form the shell.^6^ This protein assembly is further reinforced by a secondary protein, GvpC, which externally fortifies the shell, enhancing the GVs' mechanical strength.^10^ The low density and high compressibility of GVs enable effective sound wave scattering, generating substantial ultrasound backscatter.^2^ This feature becomes crucial when GVs are heterologously expressed as ARGs in genetically engineered cells,^3,4^ broadening their application from targeted cellular imaging to real-time monitoring of biological processes.^11–15^

Distinguishing GVs' backscatter from the tissue necessitates a method that separates these signals. The distinct mechanical behavior of GVs, marked by reversible nonlinear buckling beyond specific pressure thresholds, facilitates the use of amplitude modulation (AM) techniques for isolating their signals from background tissue clutter.^16–18^ AM pulse sequences typically involve three sequential transmit pulses, with one at full amplitude and two at half amplitude. Only the full-amplitude pulse is designed to be above the pressure threshold for GV buckling, resulting in a nonlinear pressure response, which is minimal in tissue. By subtracting the signal elicited by the two half-amplitude transmissions from the full-amplitude pulse, tissue clutter is minimized, whereas the nonlinear response of the GVs is amplified. This process effectively enhances the visualization of cells expressing GVs or systemically injected GVs.

AM based on cross-propagating waves (xAM) has proven especially effective in detecting GVs while minimizing artifacts arising from nonlinear wave propagation through GV inclusions.^17^ As an alternative, BURST imaging provides the most sensitive detection of GV by capturing the unique signals produced by their intentional acoustic collapse—enhancing detectability by an order of magnitude compared to AM5.^19^ However, the applicability of BURST is limited in contexts requiring preservation of the GVs, such as dynamic imaging or biosensing. Therefore, enhancing the detection sensitivity of AM-based imaging for GVs is a critical goal in biomolecular ultrasound.

Harmonic imaging has been a key approach in improving the contrast and resolution of ultrasound images. For example, tissue harmonic imaging is routinely used in diagnostic ultrasonography for generating images with superior tissue definition, improved signal-to-noise ratio, and reduced artifacts produced by side lobes, grating lobes, and reverberation.^20,21^ The principle behind harmonic imaging lies in its use of the higher-frequency harmonics generated by the nonlinear propagation of the fundamental ultrasound wave. These harmonic waves typically contain fewer artifacts compared to images produced using conventional fundamental wave ultrasound. Building on this approach, the integration of harmonic imaging with specialized pulse sequences such as AM and pulse inversion has enhanced the detection of ultrasound contrast agents.^22^ The relatively strong harmonic signals arising from the agents' nonlinear acoustic behavior help set them apart from the surrounding tissue.^23^ In fields such as cardiology and hepatic imaging, harmonic imaging provides refined views of myocardial perfusion and endocardial borders, as well as more accurate lesion characterization.^20^

In this study, we evaluate the potential of using harmonic signals to enhance the detection of GVs. While existing research suggests that GVs can produce harmonic scattering,^2,11,24^ this observation has not been integrated with AM, and previous work suggested that fundamental-frequency imaging provided the best GV imaging performance with conventional parabolic AM (pAM) sequences.^16^ We hypothesized that integrating harmonic imaging with xAM could significantly improve GV detection sensitivity due to the cleaner nonlinear background of xAM. To test our hypothesis, we imaged tissue-mimicking phantoms containing purified GVs,^8^ mammalian cells genetically engineered to express GVs as acoustic reporter genes,^5^ and live mice following systemic infusion and liver uptake of GVs.^25^ Furthermore, we compared the performance of xAM and its harmonic counterpart throughout the study.

For all experiments, we employed a 128-element linear array ultrasound probe with a nominal bandwidth of 14–22 MHz. To integrate harmonic signals with xAM imaging, we performed tests at a transmit frequency of 12.5 MHz, where we expected to observe second harmonic contributions manifesting at 25 MHz (Fig. 1). To evaluate the effectiveness of this approach, we compared the outcomes with those obtained from conventional xAM imaging, which was performed at a baseline transmit frequency of 15.625 MHz (whose second harmonic is beyond the bandwidth of the transducer). We ensured that both the 12.5 and 15.625 MHz frequencies were operated at the same transmit pressure of approximately 400 kPa, facilitating a direct and consistent comparison between the two imaging modes.

**FIG. 1.**
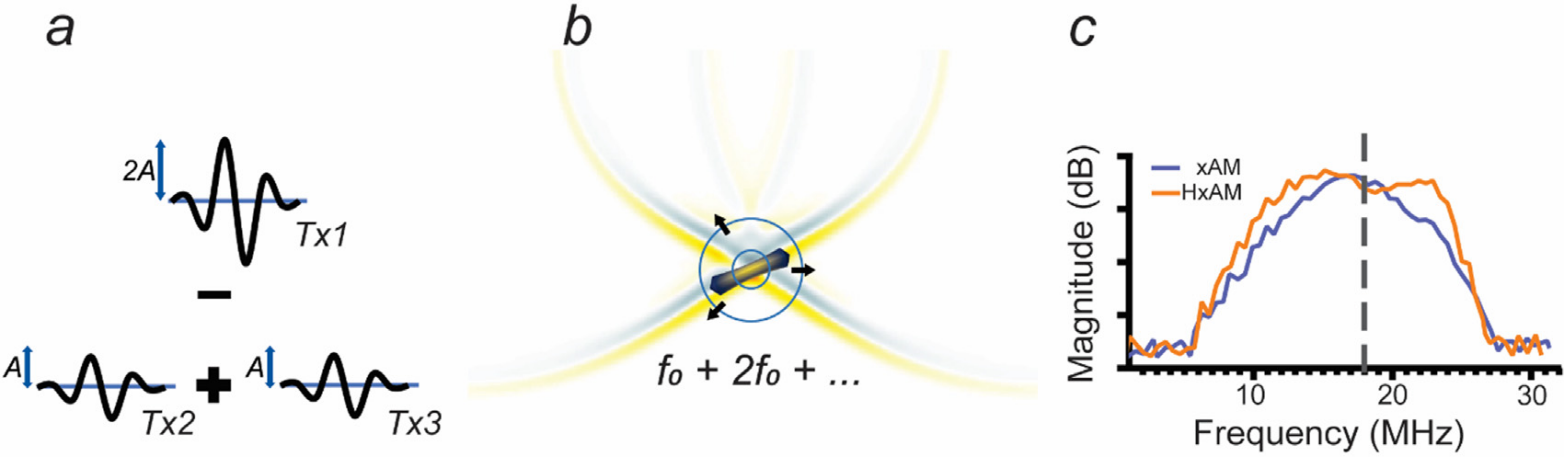
Cross-wave propagation and amplitude modulation in harmonic imaging. (a) Depiction of a three-pulse sequence utilized for amplitude modulation, where the first pulse has twice the amplitude of the subsequent pulses to induce modulation. (b) The nonlinear scattering behavior of harmonic gas GVs when sonicated above their buckling threshold, as indicated by the converging cross-waves. (c) Comparison of the receive spectra for ultrasound transmissions at 15.625 and 12.5 MHz; the latter captures the harmonic signal within the bandwidth limitations of the transducer and scanner. The dashed gray line marks the 17.5 MHz frequency threshold used for high-pass harmonic filtering, as reported in Fig. 2.

We began by imaging purified *Anabaena flos-aquae* GVs, stripped of GvpC to enable buckling, in tissue-mimicking phantoms^8^ [Fig. 2(a)]. We conducted imaging of the samples using xAM, harmonic xAM (HxAM) using the full received signal, and a variant of harmonic xAM (HxAM-f) employing a high-pass receive filter set at 17.5 MHz to exclude the fundamental signal. We assessed the efficacy of imaging techniques at varying concentrations of GVs, determined by optical density (OD) measurements, which is based on light scattering. An OD of 1, measured at a wavelength of 500 nm (OD_500_), corresponds to a concentration of approximately 184 pM GV particles.^8^

**FIG. 2.**
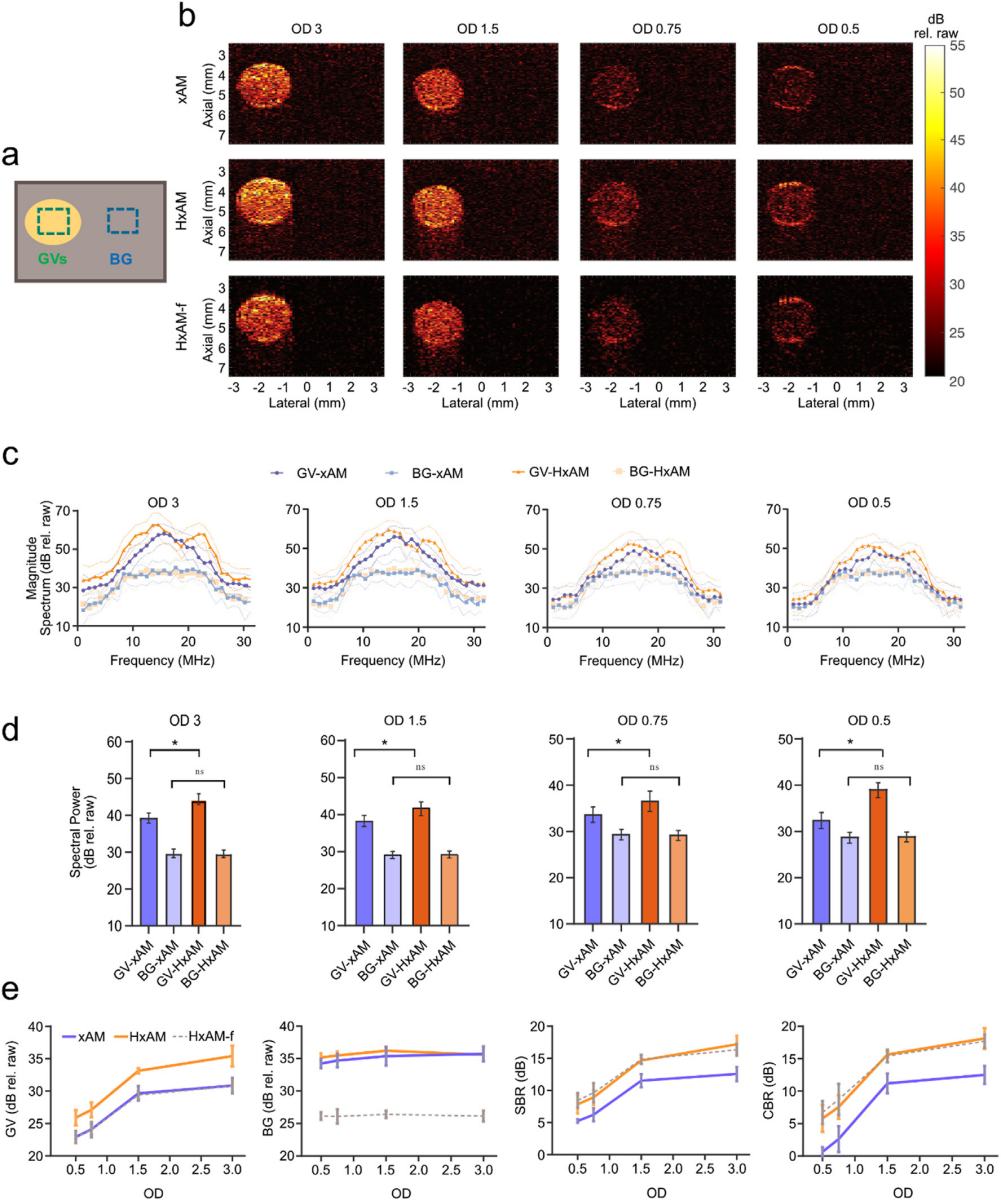
*In vitro* imaging of GVs at various concentrations in tissue-mimicking phantoms using xAM and HxAM. (a) Schematic of the ultrasound phantom with GV inclusion (yellow) in a tissue-mimicking matrix (gray). ROIs are indicated in green for GVs, and in blue for the background. The GV inclusions are positioned at a depth of 4.5 mm and have a radius of 1 mm. (b) Representative xAM (top row) and HxAM (middle row) images of the same cross section at different GV concentrations. The bottom row shows HxAM images high-pass filtered at a 17.5 MHz threshold to highlight harmonic contributions. All images are displayed using the same dynamic color range. (c) Magnitude Fourier spectra for GV and background signals in xAM and HxAM images at various GV concentrations. (d) Quantitative analysis of GV and background signals in xAM and HxAM images as related to GV concentration, including STR and CTR performance metrics. (e) Spectral power from the Fourier spectrum of GV and background signals in xAM, HxAM, and HxAM-filtered images at different GV concentrations. Data from N = 5 samples; error bars represent the standard error of the mean. Signal amplitude presented in decibels (dB rel. raw) are calculated based on the absolute signal recorded directly from the Verasonics scanner without further normalization. Statistical significance is indicated by ‘^*^', and non-significance by ‘ns'.

HxAM yielded considerably enhanced images compared to conventional xAM [Fig. 2(b)]. Spectral analysis revealed a distinct second harmonic signal at 25 MHz, which was solely attributable to the GVs and was not present in the tissue-mimicking background [Figs. 2(c) and 2(d)]. These attributes translated into a notable increase in signal to background ratio (SBR) and contrast-to-background ratio (CBR), with improvements of 4.7 and 5.6 dB, respectively. Furthermore, such enhancements stemmed from an increase in the GV signal, while background levels remained the same between the two methods [Fig. 2(e)]. HxAM maintained a performance advantage of over 5 dB compared to xAM regardless of averaging parameters. With just 15 averaging repeats, HxAM surpassed 50 such repeats of xAM in imaging quality (supplementary material Fig. 1). The resulting increase in imaging frame rate can be especially useful for *in vivo* applications and dynamic imaging.

The filtering in HxAM-f substantially suppressed background signal, while also reducing GV signal, resulting in overall SBR and CBR similar to unfiltered HxAM [Figs. 2(b) and 2(e)]. To confirm that the distinct harmonic signals observed in HxAM can be attributed to the nonlinear buckling behavior of GVs, we also imaged stiff-shelled GVs that do not exhibit robust buckling behavior^11^ due to the presence of GvpC and saw a lack of second harmonic output (supplementary material Fig. 2).

In contrast to HxAM, harmonic imaging using the parabolic focusing of pAM did not significantly increase SBR or CBR compared to fundamental imaging (supplementary material Fig. 3), consistent with our previous report.^16^ This difference arises from a lower harmonic-to-fundamental signal ratio (0.69, compared to 0.98 in xAM), which we attribute to artificially elevated fundamental signal at full transmit amplitude caused by cumulative nonlinear propagation.^17^ In contrast, xAM imaging by design has lower propagation nonlinearity, providing a higher harmonic-to-fundamental ratio for harmonic imaging and a more faithful representation of scatterer locations in the medium.

To ensure that the enhanced contrast of HxAM does not arise from differences in focal dimensions, we mapped the beam profiles for xAM and HxAM transmissions using an acoustic hydrophone. The elevational and lateral profiles of xAM and HxAM imaging showed no major differences. The full width half maximum (FWHM) values for the elevational beam profile were 0.866 mm for xAM and 0.855 mm for HxAM, while for the lateral beam profile, they were 0.806 and 0.785 mm, respectively (supplementary material Fig. 4). Although HxAM has slightly more pronounced side lobes, their amplitude remains below the GVs' buckling threshold at our transmit pressures, minimizing the likelihood of artifact generation. The increased side lobes observed when shifting from 15.625 to 12.5 MHz are primarily due to the deviation from the transducer's center frequency (18.5 MHz) and moving outside its manufacturer-recommended bandwidth (14–22 MHz). This was necessary to demonstrate proof of concept for harmonic imaging, as the existing bandwidth could not fully accommodate both fundamental and harmonic frequencies. Transducers are designed to perform optimally within their specified frequency range, and operating outside this range can degrade beam quality, leading to increased side lobes. However, this issue can be addressed by choosing a broader bandwidth transducer such as L10-4 or L35-16 that can accommodate both fundamental and harmonic frequencies.

To evaluate the utility of HxAM imaging in applications of GVs as acoustic reporter genes, we imaged MDA-MB-231 cancer cells genetically engineered to express GVs^5^ [Fig. 3(a)]. This cell line is commonly used to model breast cancer *in vivo*. HxAM improved the detection of cells across a wide range of concentrations. At the highest densities—3 × 10^6^ and 3 × 10^7^ cell/ml—HxAM increased SBR and CBR relative to xAM [Figs. 3(b)–3(d)]. At lower densities where individual cells are expected to be separated within the field of view, HxAM facilitated the identification of a larger number of signal sources (putative cells) compared to xAM—identifying 2.4 times to 3.0 times more sources [Figs. 3(b) and 3(e)]. To ensure that the contrast points contained GVs, we applied ultrasound pulses at a high pressure (3.6 MPa) using focused parabolic delays exceeding the GV's irreversible collapse threshold (∼570 kPa) and documented a loss of scattering (supplementary material Fig. 5).

**FIG. 3.**
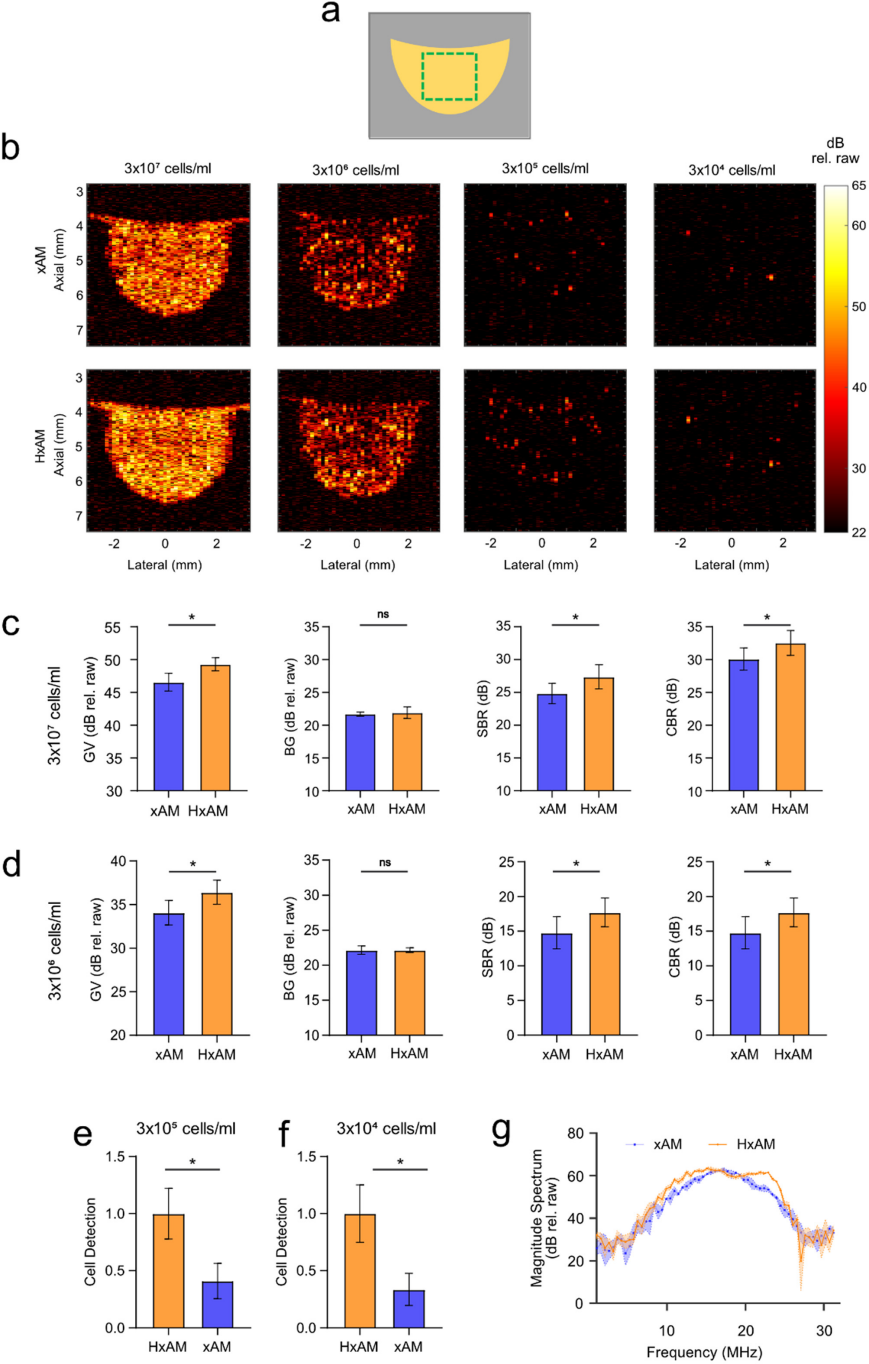
Comparative ultrasound imaging of engineered mammalian cells expressing acoustic reporter genes *in vitro*. (a) Schematic representation: Mammalian cells (in yellow) embedded within an agar phantom (gray). (b) xAM and HxAM Imaging: Images of mammalian cells genetically engineered to express acoustic reporter genes, set in agar at various cell concentrations. All images are displayed using the same dynamic color range. (c) and (d) Signal Analysis: Quantitative evaluation of nonlinear ultrasound signals from xAM and HxAM imaging at concentrations of 3 × 10^7^ and 3 × 10^6^ cells, respectively. Analysis includes assessment of gas vesicle (GV) signal from a specified region of interest (ROI) shown in (a) and background signal from the same ROI post-GV acoustic collapse. Bar graphs show GV signals with performance metrics like STR and CTR. (e) and (f) Cell count analysis: Cell counts in HxAM and xAM images at concentrations of 3 × 10^5^ and 3 × 10^4^ cells, respectively, normalized to HxAM counts at each concentration. Data based on N = 5 samples; error bars denote standard error of the mean. (g) Fourier spectral analysis: Magnitude Fourier spectra derived from xAM and HxAM imaging of a single cell, which is indicated by a blue arrow in (b). Signal amplitude presented in decibels (dB rel. raw) are calculated based on the absolute signal recorded directly from the Verasonics scanner without further normalization. Statistical significance is indicated by ‘^*^', and non-significance by ‘ns'.

To assess the efficacy of HxAM *in vivo*, we imaged mice during intravenous administration of purified GVs, focusing on the liver, where GVs are taken up and degraded as a part of the organ's phagolysosomal function.^2,25^ To evaluate the detection sensitivity, we injected 100 μl solutions containing GVs at either a standard OD of 30 or a minimally detectable concentration of OD 10 [Fig. 4(a)]. Furthermore, to enable imaging of the same exact plane with HxAM and xAM and eliminate confounds from physiological motion, we euthanized the animals immediately before imaging. HxAM allowed GV uptake to be imaged substantially deeper inside the liver tissue at both standard [Fig. 4(b)] and low GV concentrations [Fig. 4(c), supplementary material Fig. 6(a)], extending detection depth by up to 20% [Fig. 4(d)]. The GV-based source of the contrast was confirmed by collapsing the GVs with high-pressure pulses. This improvement was not due to deeper penetration of the transmit pulse; the expected difference in attenuation between 15.625 and 12.5 MHz is only 0.78 dB over 5 mm of liver tissue.^26^ Spectral analyses confirmed the presence of harmonic signals in HxAM [Fig. 4(e), supplementary material Figs. 6(b) and 6(c)]. HxAM imaging improved SBR and CTR by 4.32 and 11.1 dB at OD 30 [Fig. 4(f)], and 4.4 and 15.8 dB at OD 10 [Fig. 4(g)], relative to xAM.

**FIG. 4.**
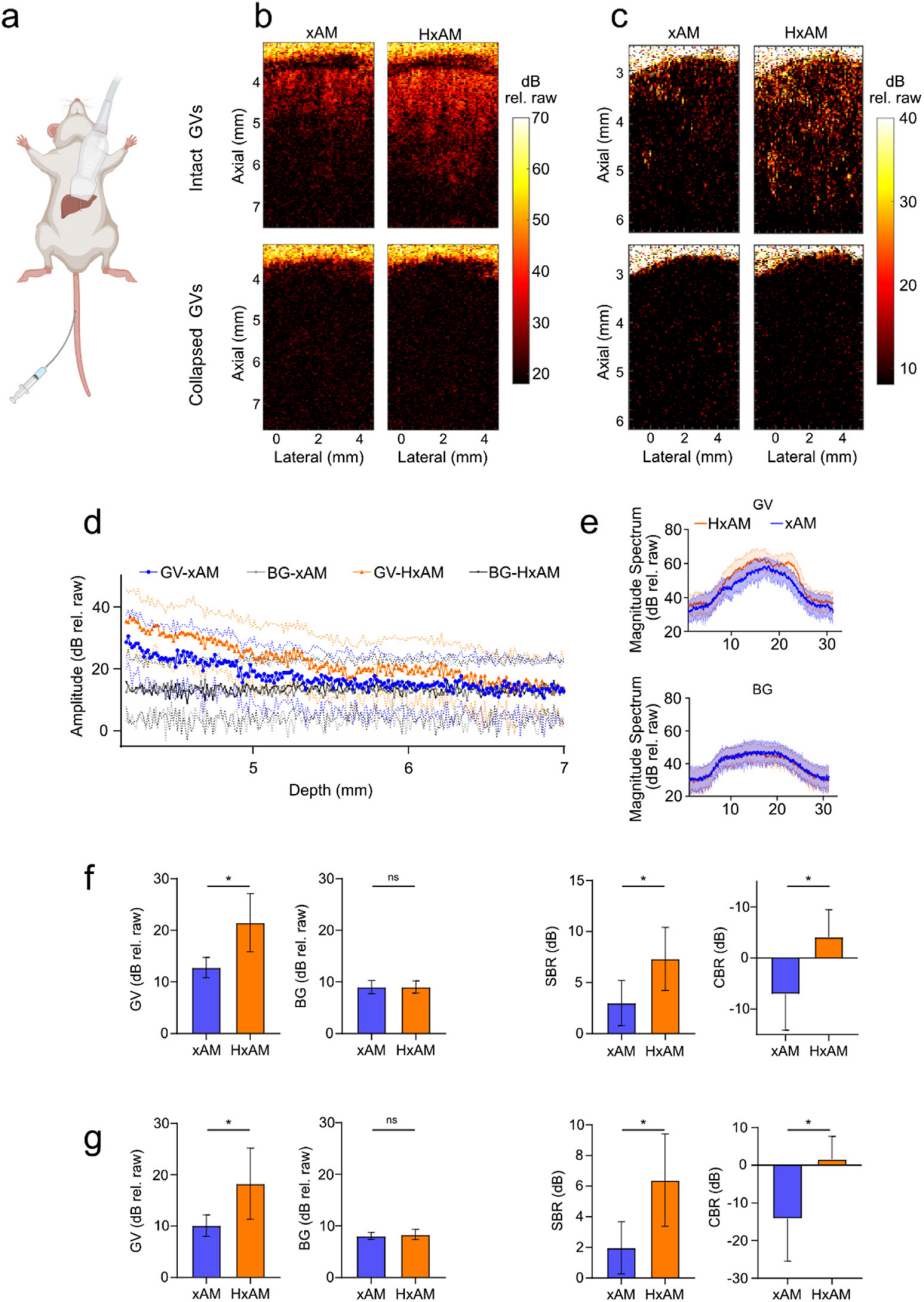
*In vivo* ultrasound imaging of mice liver after intravenous injection of purified GVs. (a) Schematic illustration of the ultrasound mouse liver experiment. (a) and (c) Representative examples of xAM and HxAM images of mice liver, acquired at the same cross section using both imaging techniques at OD_500_ 30 and 10, respectively. Top and bottom rows correspond to detection of GVs intact and after acoustic-based collapse, respectively. (d) Axial line plots corresponding to GV and background signal at varying depths of images in (b). The solid line plots and the corresponding dotted double-sided bands represent mean and the standard error, respectively, estimated across all columns of the ultrasound images in (b). (e) Magnitude Fourier spectra associated with GV and background signals in xAM and HxAM images reported in (b). (f) and (g) Quantitative analysis of GV and background signal in mice liver using xAM and HxAM imaging, at OD_500_ of 30 and 10, respectively. The negative CBR arises from taking the mean of the GV signal across the entire liver in the image, except for the superficial skin layer. N = 5 mice at each GV concentration, and the error bars represent standard error of the mean. Signal amplitude presented in decibels (dB rel. raw) are calculated based on the absolute signal recorded directly from the Verasonics scanner without further normalization. Statistical significance is indicated by ‘^*^', and non-significance by ‘ns'.

Taken together, the results of this study suggest that HxAM imaging improves the detection of GVs over xAM in all the main *in vitro* and *in vivo* scenarios. Harmonic frequencies are selectively amplified in the GV signal without altering the background, leading to marked improvements in SBR and CBR. We anticipate that this enhanced sensitivity will facilitate *in vivo* cell imaging, while its specificity for buckling GVs over stiff GVs will contribute to dynamic biosensing.^12,27^ The enhanced performance of HxAM is attributed to the capture of harmonic signals arising from GV scatterers, with minimal background from nonlinear propagation artifacts (unlike pAM). Even though the current experiments were performed with imaging depth up to 8 mm using a high-frequency probe, we anticipate that the imaging depth can be extended by using a lower imaging frequency transducer, such as used to image GVs in a previous study.^5^

HxAM imaging inherits some limitations of xAM imaging, including a reduced lateral and axial field of view. It is also important to note that the frame rate of HxAM is identical to that of xAM. We have successfully used xAM in several previous *in vivo* studies^5,12,17,25^ without encountering any frame rate-related issues. However, we acknowledge the potential impact of motion on multi-pulse techniques, and in future studies, we plan to explore the extension of harmonic imaging to ultrafast imaging sequences to further reduce any motion-related challenges. Furthermore, HxAM imaging adds the requirement for transducers and scanners with relatively high transmit-receive bandwidth. However, HxAM's improved ability to detect GVs and GV-expressing cells makes it an attractive imaging method for biomolecular and cellular ultrasound.

## METHODS

### Ultrasound acquisition sequence

We use a Verasonics Vantage ultrasound scanner, equipped with an L22-14vX probe, to execute various imaging sequences such as xAM, pAM, and its respective harmonic versions. This probe features a linear array composed of 128 elements, each spaced at a 0.10-mm interval. It has an elevation focus of 8 mm, a 1.5-mm elevation aperture, and operates at a central frequency of 18.5 MHz, offering a bandwidth of 67% at −6 dB. For transmission, we use single-cycle waveforms at frequencies of 15.625 and 12.5 MHz for each active element in the array, which ensures that our base frequency is divided by factors of 4 and 5, respectively, in sync with the system's 62.5-MHz sampling rate. For harmonic versions of xAM and pAM, we opt for a transmit frequency of 12.5 MHz, while maintaining a receive sampling rate of 62.5 MHz—the highest rate achievable on the Verasonics Vantage system. To balance lateral view and axial depth, the xAM sequence employs an aperture consisting of 65 elements, centering the array but silencing the middle element to allow for symmetric AM coding. This setup results in 64 ray lines for each xAM image. For the pAM sequence, we adjust the focal depth of the parabolic delays to 4.5 mm, aligning it with the center of the GV cylindrical inclusion. We use a 38-element aperture for this sequence, resulting in an F-number of 1.125 and producing 89 ray lines to optimize the dimensions of the pAM images. The raw radio frequency (rf) data are collected and processed through a custom-built, real-time image reconstruction pipeline, as described in the paper.^17^ This includes a specialized beamforming algorithm tailored to meet the unique demands of xAM imaging. Furthermore, the signal to noise ratio (SNR) of the acquired radio frequency (RF) data were enhanced using repeated transmit-receive events, which involves accumulating the acquired data at the same receive location within the dRAM memory to suppress background noise in the acquired signals. We selected 15 averaging repeats as the standard for both HxAM and xAM imaging techniques, as per experiments and analysis performed in Fig. 1 of the supplementary material.

Finally, for both xAM and HxAM imaging techniques, the received RF data corresponding to the three AM transmit sequences were accumulated at the same location in the receive buffer of the scanner. We applied an apodization of −1 to each of the two half-amplitude receives of the AM sequences, aiding in the process of AM cancelation. The reasoning behind aggregating the received data from the three different transmit sequences at a single location, as opposed to collecting them at separate locations and then subtracting them during post-processing, was to ensure there was no saturation of the receive buffer, which has a 14-bit dynamic range. This approach was particularly crucial when working with a higher number of averaging repeats. If the buffer became saturated, especially in the data corresponding to the full amplitude transmit of the AM sequence, it could lead to imbalances in AM cancelation, which in turn would result in considerable image artifacts.

### Preparation of gas vesicles

Gas vesicles were isolated from *Anabaena sp.* as detailed in prior studies.^8^ To strip the native GVs, a 6M urea solution was applied, followed by repeated rounds of centrifugation for flotation and subsequent removal of the subnanat, yielding stripped GVs. The medium was then exchanged through four rounds of dialysis in PBS to guarantee the thorough removal of the native GvpC layer. The concentration of GVs was quantified by measuring the optical density at 500 nm (OD_500_) using a NanoDrop ND-1000 spectrophotometer (Thermo Fisher Scientific, Waltham, MA, USA). For *Anabaena sp.*, an OD_500_ of 1 is equivalent to a GV concentration of 184 pM or a volume fraction of 0.04% in an aqueous suspension.^8^ This quantification is based on the principle that purified GVs suspended in PBS scatter visible light, allowing their concentration to be accurately measured by assessing the OD at 500 nm.

### In vitro imaging of tissue-mimicking phantoms

Tissue-mimicking phantoms were prepared using a mixture of 1% agarose by weight/volume (w/v) in PBS and 0.2% AlO_3_ (w/v).^16–18^ Specialized 3D-printed molds were utilized to form cylindrical wells with a diameter of 2 mm. GVs were briefly heated at 42 °C for one minute and subsequently combined at an equal ratio with low-melt agarose that was also at 42 °C. This mixture was then loaded into the phantom wells to achieve various GV concentrations corresponding to optical density values of 3, 1.5, 0.75, and 0.5 at a wavelength of 500 nm. The AlO_3_ level was specifically selected to emulate the echogenic scatter properties of the GVs, as determined by the contrast-to-noise ratio in B-mode ultrasound imagery. The center of these GV-filled cylindrical wells was positioned at a 4.5 mm depth. All these phantoms were imaged while positioned on an acoustic-absorbing material and submerged in PBS. All phantom experiments were conducted across N = 5 samples, for different ODs and GV types.

### In vitro imaging of engineered MDA cells expressing acoustic reporter genes

For all *in vitro* cellular imaging experiments, we used MDA-MB-231-mARGAna cells cultured in DMEM supplemented with 10% TET-free FBS and 1X penicillin/streptomycin.^5^ For xAM and HxAM imaging of MDA-MB-231-mARGAna cells suspended in agarose phantoms, cells were cultured in T225 flask with 30 ml media. Cells were seeded and induced with 1 *μ*g/ml doxycycline after an overnight incubation and at subsequent days as indicated, except for the uninduced control which was grown in a 10 cm dish without doxycycline. Media was changed daily thereafter until cell harvest. Cells were trypsinized with 6 ml 0.25% trypsin/EDTA for 6 minutes at 37 °C, after which the trypsin was quenched by addition of 8 ml media. Cells were harvested and resuspended at 60 000 000 cells/ml. 10-fold serial dilutions were performed with each cell line. Each cell dilution was mixed 1:1 with 2% low-melt agarose before loading into agarose phantom wells (five replicates each). Cells were imaged with an L22-14vX transducer at 0.5 MPa for both xAM and HxAM imaging.

### In vivo imaging of mice liver

The *in vivo* experiments were performed on Cg-Foxn1^nu^/J female mice (Jackson Laboratory) aged 7 weeks under a protocol approved by the Institutional Animal Care and Use Committee of the California Institute of Technology. This study did not require any randomization or blinding techniques. The mouse was sedated using a 2%–3% isoflurane anesthesia. The experimental procedures involved administering an intravenous injection of 100 *μ*l of a sterilized GV solution dissolved in PBS.^25^ We tested two distinct concentrations of GVs, with OD_500_ of 10 and 30, examining a total of five animals for each GV concentration. After waiting for 25 min to allow for liver phagocytosis of the GVs, as supported by previous research, the animal was then humanely euthanized. Subsequently, we obtained xAM and HxAM images right away. Thereafter, we collapsed the GVs using high acoustic transmit pressure (>3.5 MPa) and reacquired xAM and HxAM images at the same liver cross section. The reason for performing imaging post-euthanasia was to eliminate any physiological motion from breathing or heartbeat. Such motion could introduce variability in the imaging cross section, thus compromising the reliability of the comparison between xAM and HxAM images.

### Quantitative and statistical analysis

Quantitative numbers to assess performance of the AM imaging techniques were computed using SBR (dB) = 20*log10 $\left(\frac{\mu_{GV}}{\mu_{BG}}\right)$ and CBR (dB) = 20^*^log10 $\left(\frac{\mu_{GV}-\mu_{BG}}{\sigma_{BG}}\right)$, where *μ* and σ denote mean and standard deviation, estimated in the identified ROIs. The Fourier spectra for both GV and BG signals were calculated using the same ROIs as those applied for other quality metrics. Columns within each ROI and across various samples were collectively aggregated as repeated measures for estimating the frequency content. The Fourier spectra for these aggregated column data were computed using the “fft” function in MATLAB. These analyses were performed for all *in vitro* and *in vivo* experiments involving tissue-mimicking phantoms, engineered cells, and mice.

Additionally, due to the sparse distribution of cells at the lower two concentrations depicted in Fig. 3, it was impractical to estimate the SBR and CBR. Therefore, we quantified pixels exceeding a specific threshold as contributing to cell presence for each imaging method. We confirmed that the threshold value resulted in a zero cell count in post-collapse images. The cell counts obtained via xAM were then normalized against those acquired through HxAM, with these comparative results reported in Fig. 3. Finally, to assess the statistical significance of the comparisons between xAM and HxAM imaging, we employed t-tests. Statistical significance was established at a p-value greater than 0.05, denoted by an asterisk (*). The dynamic ranges for the images in the manuscript were selected based on the signal strengths and specific imaging targets, with the goal of maximizing the contrast between GVs and the background. These ranges were adjusted according to the concentration of the samples in each study (*in vitro, in vivo*, and cell studies) to optimize visualization and ensure accurate representation of the data for each experimental context.

## SUPPLEMENTARY MATERIAL

See the supplementary material for additional experimental data that support the primary findings reported in this study, which includes the following:

1. Impact of averaging repeats on xAM and HxAM imaging: An analysis of how varying the number of averaging repeats impacts image quality, demonstrating that HxAM consistently outperforms xAM regardless of the number of repeats.
2. Harmonics imaging of wild type GVs: A validation study showing that wild-type gas vesicles (wtGVs), which lack nonlinear buckling, do not enhance HxAM imaging, as expected.
3. Harmonics imaging using parabolic AM pulse sequences: An *in vitro* assessment comparing parabolic AM (pAM) and xAM pulse sequences, in the context of harmonic imaging and its impact on imaging performance.
4. Elevational and lateral beam profile measurement: Beam profile measurements conducted using a fiber-optic hydrophone, detailing the beam characteristics for both xAM and HxAM imaging.
5. *In Vitro* imaging of engineered MDA cells expressing acoustic reporter genes: Ultrasound imaging of engineered MDA cells post-GV collapse, demonstrating that advantages of harmonic imaging disappear in the absence of GVs.
6. *In vivo* imaging of mice liver: Additional data from imaging of intravenously injected GVs in the mouse liver, highlighting the distinct harmonic signals observed in HxAM images.

## Data Availability

The data that support the findings of this study are available from the corresponding author upon reasonable request.
